# The Coverage Problem in Video-Based Wireless Sensor Networks: A Survey

**DOI:** 10.3390/s100908215

**Published:** 2010-09-02

**Authors:** Daniel G. Costa, Luiz Affonso Guedes

**Affiliations:** 1 DCA-CT-UFRN, Campus Universitário, Lagoa Nova, Universidade Federal do Rio Grande do Norte, 59072-970 Natal RN, Brazil; E-Mail: affonso@dca.ufrn.br; 2 DTEC-UEFS, Av Transnordestina, S/N, Novo Horizonte, Universidade Estadual de Feira de Santana, 44036-900 Feira de Santana BA, Brazil

**Keywords:** video-based wireless sensor networks, the coverage problem, directional sensing, sensor deployment, coverage metrics

## Abstract

Wireless sensor networks typically consist of a great number of tiny low-cost electronic devices with limited sensing and computing capabilities which cooperatively communicate to collect some kind of information from an area of interest. When wireless nodes of such networks are equipped with a low-power camera, visual data can be retrieved, facilitating a new set of novel applications. The nature of video-based wireless sensor networks demands new algorithms and solutions, since traditional wireless sensor networks approaches are not feasible or even efficient for that specialized communication scenario. The coverage problem is a crucial issue of wireless sensor networks, requiring specific solutions when video-based sensors are employed. In this paper, it is surveyed the state of the art of this particular issue, regarding strategies, algorithms and general computational solutions. Open research areas are also discussed, envisaging promising investigation considering coverage in video-based wireless sensor networks.

## 1. Introduction

In the last decade, wireless sensor networks (WSNs) were one of the main research topics in computer communications. Composed of low-cost powered-restricted devices with sensing and computing capabilities which cooperatively communicate in a wireless manner, WSNs allowed a variety of innovative applications for sensing in wide, hostile or even hard access areas, as in battlefield surveillance, environmental monitoring, rescue operations, home entertainment and pollution detection, among others [1]. To achieve such applicability, many aspects of these networks, ranging from energy efficiency to sensor deployment and mobile communications, have been addressed in numerous research projects [2].

Nodes in WSNs are disposable electronic devices equipped with a transceiver, an energy supply (typically a battery) and a sensing unity, although others modules can also be found [3]. In traditional WSNs, sensors collect scalar data such as humidity, temperature, pressure and seismic variations [4]. When inexpensive low-resolution cameras are embedded in wireless sensors, visual data can be retrieved from the environment too, allowing a new scope of applications. For the resulting Video-based Wireless Sensor Networks (VWSNs), or simply Visual Sensor Networks (VSNs), new researches had to be conducted, since many traditional WSN algorithms, architectures and computational solutions are not feasible or even efficient for that specific communication scenario [5,6].

A crucial point in WSNs is the coverage problem [7]. The coverage concept is subject to a wide range of interpretations. Coverage can be formulated based on the subject to be covered, the sensor deployment mechanism, the network connectivity and energy consumption [8,9]. All these issues will be surveyed in this paper, particularly considering VWSNs.

Typically, sensors will be randomly scattered in a target area, what can result in regions densely or sparsely covered by sensor nodes. For many applications, the quality of the deployed sensor network will be a direct function of how well an area of interest is covered by the sensor nodes. Maximum coverage as a result of optimal placement of sensor nodes is only feasible when deterministic deployment is considered. Others important issues directed related to the coverage problem are connectivity and energy preservation. Wireless sensor networks, no matter the kind of sensors employed, are energy constrained. To maximize the network lifetime, the role of each wireless node has to be efficiently set along the time, since it can be sensing, relaying other nodes packets or sleeping for energy saving [10]. In a densely deployed region, redundant wireless nodes can be turned off to save energy, letting remaining neighboring nodes with sensing and/or routing functions. When a wireless node fails due to energy depletion or physical damage, a sleeping node is turned on, potentially prolonging the lifetime of the network and preserving the coverage of a region. On the other hand, nodes with low energy can be selected for sensing, while wireless nodes with more energy are elected for routing, or the opposite, depending on the algorithms employed and the application requirements.

In traditional WSNs, different aspects of the coverage problem have been addressed by many works. For example, in [7] polynomial time algorithms are presented to determine if a set of wireless nodes can properly cover a target area. This problem is expanded in [11], which regards a three-dimensional sensing model. The 3D sensing range of the nodes is calculated by a low-complexity algorithm, in polynomial time. The protocol proposed in [12] preserves nodes with higher importance for the sensing application, electing them for sensing instead of routing in sparsely covered areas.

Due to the nature of directional sensing traditional solutions for the coverage problem should not be employed for video-based wireless sensor networks. Scalar sensors (also known as isotropic sensors) collect raw data from their vicinity, while cameras can capture images of close and even distant targets/scenes, resulting in a particular notion of sensing range. A reasonable discussion of the problems of using traditional coverage algorithms in VWSNs is offered in [13].

Several papers can be found in the literature surveying wireless sensor networks [2,3,14] and visual sensor networks [6,15] as their main research areas. A specific survey on the coverage problem in traditional wireless sensor networks can be found in [16]. In a different way, in this survey we present the recent developments, challenging issues and open research areas of the coverage problem in video-based wireless sensor networks. This crucial part of VWSNs is surveyed in a structured way, comprising directional sensing, node deployment, coverage metrics and energy efficient solutions, besides complementary issues related to directional coverage.

The rest of the paper is organized as follows. Section 2 provides a short description about the main concepts of VWSNs. In Section 3, the fundaments of directional coverage are presented. Deterministic deployment of video-based sensors is discussed in Section 4, along with the resulting coverage of optimal camera and sensor placement. Algorithms for coverage management, coverage metrics and node localization in randomly deployed VWSNs are presented in Section 5. Section 6 brings algorithms and strategies for coverage, connectivity and energy preservation. Section 7 presents others relevant issues in the coverage problem. Open research areas are discussed in Section 8. At last, conclusions and references are presented.

## 2. Video-based Wireless Sensor Networks

**Figure 1.**
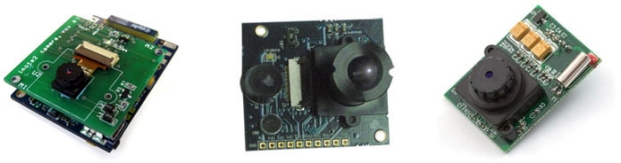
Examples of typical low-resolution cameras.

Recent advances in CMOS technology have allowed the development of low-power cameras that can be embedded in wireless nodes for a whole new set of sensing functions. Inexpensive visual sensors could be developed by the reduction of the hardware to a single integrated chip that can capture and process an optimal image [15]. Sensors equipped with a CMOS camera can collect visual data, attending applications unassisted by Internet and traditional wireless sensor networks technologies. The resolution, viewing angle and cost of video-based sensors can significantly differ depending on the application type and the network budget. Figure 1 presents some typical low-resolution cameras for visual sensor networks.

VWSNs are an emerging *ad-hoc* network technology that employs autonomous wireless sensors equipped with a low-power cameras to wirelessly retrieve visual data from the monitored field. In the last years, the demand for VWSN applications has significantly increased, fostered by vision-based applications as traffic monitoring and surveillance. For an increasing group of applications, scalar data collected from traditional wireless sensor networks are insufficient, even if a large number of sensors are deployed [5]. Other promising applications for video-based wireless sensor networks are environment monitoring, wildlife observation, automated assistance for elderly and disabled people, person location service and industrial process control [15].

Besides the use of some well-known algorithms and strategies from traditional wireless sensor networks, VWSNs demand new solutions for challenging questions, due to their particular sensing operations. For traditional WSNs, coverage and connectivity are coupled issues, since the wireless sensors collect data from their vicinity [5] and a single area is likely to be monitored by neighboring nodes. Thus, for visual sensor networks, a different concept of sensing range is created [14]. As cameras can capture images of close and even distant targets/scenes, two sensors with no physical proximity can retrieve a similar image, demanding new computational solutions for handling the coverage problem.

Others relevant issues emerge when the wireless sensors are equipped with cameras. Given the large amount of data generated by camera nodes, data transmission requires more bandwidth (and energy) in VWSNs. To reduce the amount of data transmitted, many works suggest and propose algorithms for local processing of visual data. Data aggregation is addressed as a crucial point in video-based wireless sensor networks, requiring complex and sometimes costly algorithms to process visual data, when compared with scalar data provided by traditional WSNs [17]. In fact, video-based wireless sensor networks may expect more energy consumption in local processing than in data transmission, contrary to traditional wireless sensor networks [6].

In addition, the nature of visual data imposes time constraints to the multi-hop communication among wireless nodes. Therefore, new protocols and network topologies were created or adapted to couple with the transmission of such data type [18]. Moreover, QoS has been pointed as a valuable resource for video-based wireless sensor networks [15].

Multimedia in-network processing is another key design requirement of VWSNs, addressed by many works [15]. It is expected cooperative processing of multimedia data by intermediate nodes, potentially reducing the amount of data transmitted throughout the network and prolonging the overall network lifetime by saving energy, since the communication latency is kept in an acceptable level.

Many others relevant issues are associated to video-based wireless sensor networks, comprising a diversity of interdisciplinary aspects with particular challenges. However, this work is focused on the coverage problem in VWSNs, which have a direct impact in the quality of the deployed network and the user application, but also is influenced by many aspects as node deployment and energy preservation approaches.

## 3. Fundaments of Directional Coverage

The coverage is a crucial issue directed related to the final quality of the application, also impacting in the way *ad-hoc* networks operate. In fact, for most WSN applications, how well a monitored field is covered is a fundamental concern that should be properly addressed. But such concern varies according to the nature of the applications. For example, for a surveillance application, the lack of monitoring of an area of 1 m × 1 m can deplete the application quality, since an object may not be detected in the uncovered area. However, such restriction cannot be so strict for some kind of applications, which are concerned with the sensing of large areas, as in weather monitoring, where there is no need for collecting data from every single subspace of the field.

When dealing with coverage, we wish to determine how well an area of interest is covered by wireless nodes and how redundant nodes can be used to prolong the network lifetime, keeping a minimum level of coverage quality (required by the application) and connectivity of the nodes. For these challenging issues, a sensing model has to be created according to the way sensors collect date from the monitored field.

In traditional wireless sensor networks, the sensing range of wireless nodes can be approximated to the radius of a circumference [7]. Scalar data is collected according to the type of the sensor, its accuracy and the sensing range. For this sensing structure, neighboring nodes are likely to collect similar data. For traditional WSNs, the sensing and connectivity scopes are equivalent, associated to the nodes’ vicinity.

For visual sensor networks, video-based sensors collect data in a different way, creating a directional sensing model. Additionally, some aspects of digital cameras, such as lens quality and zoom capabilities, can influence the final sensing and coverage. Although there are many options and configurations, cost will often limit the quality and additional features of video-based sensors.

The maximum volume visible from a camera is defined as the Field of View (FoV). It is a sector-like visible region emanating from the camera [13], defining a direction of viewing (camera’s pose). The spatial resolution of a camera is the ratio between the total number of pixels used to represent an object and its size. More detailed images are directly proportional to higher spatial resolution [19], but with direct influence in the final cost of the camera. The Depth of Field (DoF) is the amount of distance between the nearest and farthest targets that can be properly viewed by a camera [19]. Due to limited resolution and distortion of lenses, cameras in real-world VWSNs have a limited depth of field [20]. In fact, targets too far from the optical center may not be observed. The viewing angle is the maximum angle at which an object of the scene can be observed [19]. As each camera has a viewing direction, it is very acceptable to conceive that each sensor in a VWSN has a unique perspective of the monitored field [13].

**Figure 2.**
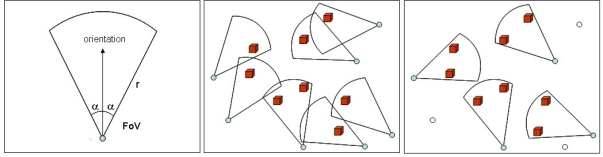
Directional sensing model. **(a)** A simple representation of cameras’ FoV; **(b)** Seven sensors covering eight targets; **(c)** Changing cameras’ orientation for a more efficient coverage.

Figure 2(a) presents a simple graphical 2D representation of a typical camera’s field of view. The viewing angle is “2α” and “r” is the sensing radius. The orientation of the cameras’ field of view is a key parameter for network coverage. Figure 2(b) shows a configuration where seven sensors are employed to cover eight targets. Changing the cameras’ orientation also change their covered area, as depicted in Figure 2(c). The same eight targets are now covered by only four sensors, reducing the cost of the deployed network and allowing the using of redundant nodes to prolong network lifetime. Note that only the cameras’ orientations were changed, not their positions.

When video-based sensors are deployed, others important issues have to be considered. When the FoV of two or more cameras intersect, the same object or scene is viewed by more than one wireless node, even under different directions and perspectives. This overlapping area can be processed for image compression or utilized for localization and optimal deployment purposes. On the other hand, the occlusion occurs when the field of view of a camera is blocked by some obstacle, which can be statically positioned or moving across the monitored field. In a modeled monitored field, a prohibited area is a region where moving or static objects cannot be placed (e.g., inside a wall). When computing coverage, such regions may not be processed, saving time and computational resources.

The coverage in VWSNs can be also influenced by the type of the cameras. A frequently deployed camera has an unchangeable fixed position and orientation. Besides fixed cameras, video-based wireless sensor networks can be deployed using adjustable cameras. A typical adjustable camera has a PTZ (Pan-Tilt-Zoom) capability, which can rotate around their horizontal and vertical axis [19], as well as manage their focal length.

For video-based wireless sensor networks, the concept of vicinity is valid only for communication, since the sensing range in WSNs is replaced in VWSNs by the field of view. In fact, two wireless nodes can collect visual data from the same object/scene, even if they are many hops away from each other. However, a very close object can be out of the FoV of a camera, what would be not true to an active wireless node in traditional WSNs. Other challenging issues are overlapping, occlusion and node redundancy, specially influenced by the type and quality of deployed cameras.

**Figure 3.**
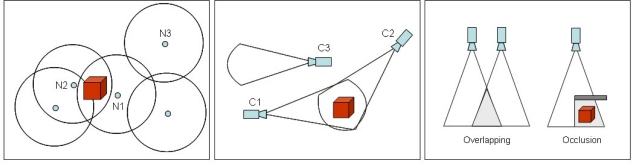
Sensing in WSNs and VWSNs. **(a)** Traditional sensing in WSNs; **(b)** Directional sensing in VWSNs; **(c)** Overlapping and occlusion.

Figure 3(a) exemplifies the sensing range in traditional wireless sensor networks. N1 and N2 are neighboring sensor nodes that are sensing the same target. N3 cannot sense the monitored target, since that object is out of the N3’s sensing range.

A different sensing model is presented in Figure 3(b). C1 and C2 are video-based sensors that can view the same object (from a distinct perspective), even though they are far away from each other. On the other hand, C3 cannot view the monitored target, even considering it is nearer from the target than the other two video-based sensors. In Figure 3(c) overlapping and occlusion situations, using a triangular FoV simplification are graphically presented. All these figures present an approximated 2D model, which can be slightly different from real-world sensing range.

## 4. Coverage in Deterministic Deployment

Video-based wireless sensor networks can be deployed in two distinct ways. In the deterministic deployment, video-based sensors are neatly placed following a pre-processed plan. In this approach, the coverage is maximized with a minimum number of sensors, reducing the final cost of the sensor network [21]. In random deployment, on the other hand, sensors are scattered in the monitored field, typically with no planning or previous knowledge of the region. For example, wireless sensors can be dropped from an airplane, over a hostile or hard-access area [21]. The deterministic deployment in VWSNs and its relation with the network coverage will be surveyed in this section, leaving the discussion of random deployment for Section 5.

Each type of application demands a specific deployment approach. For example, video-based wireless sensors can de deployed on the ceiling of an airport, for human tracking and monitoring. Others applications expect wireless nodes randomly placed in the monitored field, since determinist deployment can be not feasible in wide open areas or hard-access regions. In fact, how wireless sensors will be deployed strongly impacts the final coverage of the network.

The deterministic deployment can be divided in two groups. In static/offline deployment, the deployed elements (traditional sensors, video-based sensors, cameras, *etc.*) cannot change their position after deployment [22]. The algorithm for coverage optimization is executed only once, typically in a centralized computer. If the element can change their position or their FoV, it is performed a dynamic/online deployment. In such case, the optimal coverage has to be recalculated along the time, since the optimality of the initial positions may become void during the operation of the network, and the sensors allow a new configuration of their coverage sensing areas [22].

Many previous works have been concerned with planning of sensor positioning, most of them in the computational geometry field. When visual sensor networks emerged, camera coverage became a major issue, requiring new research focused in the challenges imposed by the using of low-resolution cameras embedded in tiny battery-operated wireless sensors. The works in computational vision provided a basis for coverage processing in sensor network area.

### 4.1. Optimal Camera Placement

Optimal camera positioning is a complex area that has led to many works in the last decades [23]. Although most of the research on optimal camera placement found in the literature does not regard energy, processing and connectivity constraints, which are key aspects of video-based wireless sensor networks, their contributions influence coverage investigation in modern VWSNs.

The camera placement is a design problem directed associated to the coverage of a region and the final quality of an application, as surveillance and human tracking. The minimum number of cameras, the camera types, their physical position, cameras’ orientations and placement density are main deployment parameters which should to be discovered and optimized. In short, we wish to answer three fundamental questions: How many cameras/sensors do we need? Of what type? Where should they be placed?

Some works initially investigated the problem of optimal camera placement. In the “art gallery problem”, we wish to know the minimal number of observers (e.g., cameras) and their static positions so that every point in a gallery room can be viewed by at least one observer [24]. This problem proved to be NP-hard in 3D modeling, fostering the development of approximated algorithms. For the “floodlight illumination problem”, it is desired to optimize the illumination of planar regions by light sources [25]. Additional works investigated visibility optimization in applications as surveillance and mobile targets tracking [26–28]. The “next best view problem” is another interesting viewing problem concerned with the minimal and optimal acquisition of images to cover an area [29,30]. Such computer vision problems are relevant for coverage calculation and optimization in video-based wireless sensor networks, but still demand additional computational solutions and novel research to deal with *ad-hoc* battery-constrained wireless sensor cameras. Many previous works in computer geometry are highly theoretical, making unrealistic assumptions such as unlimited field of view for cameras and highly controlled indoor monitoring [19].

In recent years, many works have further investigated the problem of optimal camera placement, but considering more realistic assumptions. Their results and conclusions bring significant contributions for coverage research in VWSNs.

In [31], Mittal and Davis focus on the deterministic placement of cameras in a dynamic and occluded scene, aiming at optimal positioning with the minimal number of cameras to cover an area of interest, assuming cameras with unchangeable orientation after deployment. It is considered the presence of obstacles (trees, furniture, columns, moving people, *etc.*) that generate occlusion in cameras’ FoV and may interfere in the final coverage of the monitored field. The authors also investigated worst-cases scenarios, regarding targets moving in a non-cooperative way. To calculate an optimal coverage, it was proposed a stochastic algorithm that uses a probabilistic method to analyze the visibility of the cameras, where the probability of an object to be viewed is calculated according to many constraints (physical position, field of view, resolution, prohibited areas, *etc.*). The optimal configuration is the one that reduces the cost function defined in that work.

A similar research is taken in [19], where is presented a method for positioning of static cameras. Additionally to the analyses conduced in [31], task-specific requirements and real-world constraints, as camera costs and network budget, are taken in account. An additional assumption is that type and quality of deployed cameras can be chosen by the application, while [31] works with homogenous cameras. The proposed algorithm for this camera placement problem employs a binary optimization technique, considering the sensed environment as a discrete area mapped in a polygon with possible holes.

Optimal camera placement is also investigated in [32,33]. A key concept of these works is cost restrictions, presenting solutions for minimally required coverage for low-cost networks, as well as maximum coverage when cost is not a concern. They also regard a given sampling frequency as input parameter for the camera optimal placement. In [32], the monitored field is modeled as a grid, an interesting idea that appears in many works. For Hörster *et al.* [33], two different approaches for optimal camera calculation are investigated. For precise calculations, linear programming is employed. When energy and processing time have to be spared, a reasonable solution is to use heuristics. The proposed algorithms also regard monitored fields having arbitrary shape, in contrast to solutions that consider polygonal regions [31].

The work in [34] also investigates nodes placement, trying to determine the optimal number of cameras/sensors and their optimal positioning for a convex area. However, different types of multimedia sensors are studied, not only cameras. The desired surveillance tasks and performance restrictions are additional parameters for the optimal placement calculation. Some interesting conclusions of this work are that two cameras in a square region should be placed in diagonals, in order to increase the covered area with reduced overlapping. Additionally, experiments in [34] showed that cameras with FoV above 45° do not increase the camera performance in the proposed solution.

An optimal configuration of cameras/sensors is also discussed in [35]. A general visibility model is proposed, solving the optimization problem using a Binary Integer Programming (BIP) approach, as in [34]. A more realist camera model is considered, with self and mutual occlusion issues, in 3D environments. In [36] it is also proposed a camera placement strategy using an iterative grid based binary integer programming.

In [37], the authors wished to discover the worst-case coverage in a network of cameras. In a polynomial time, the proposed algorithm calculates the maximum distance that a mobile element can be of a camera to be viewed. The presented results can help in finding areas with no coverage in tracking applications.

Cameras with wide field of view can potentially expand the covered area and avoid undesired overlapping. In [38], the optimal placement of cameras with 360° viewing is investigated. The art gallery problem is treated using such type of camera in the vertices of the polygon that comprises the monitored area. The proposed algorithm to solve this problem requires less interaction than solutions with traditional cameras.

Wide cameras’ field of view is a key aspect of the work presented in [39]. Using cameras with fisheye lenses can reduce overlapping and increase the coverage of the deployed cameras. Gonzalez-Barbosa *et al.* [40] employ both directional and ominidirectional cameras for optimal coverage. The authors argue that a hybrid approach can reduce the overall cost and maximize the coverage of the deployed cameras.

Large viewing angle [39] of even 360° viewing [38,40] can change the directional sensing model, approximating to the ominidirectional sensing of traditional WSNs. However, due to cost and energy restrictions, low-resolution cameras embedded in wireless sensors will typically have a limited viewing angle, ranging from 30° to 60° [15,21,45].

Most previous works discussed in this subsection considered deterministic placement of cameras that cannot change their current orientation after deployment. However, for dynamic scenes, deterministic placement of static cameras following a predefined plan can result in suboptimal coverage, when mobile targets have to be viewed or obstacles are moved. For these particular scenarios, strategies for self-configuration of camera’s field of view can be applied for dynamic/online coverage optimization. Only for comparison purposes, an interesting use of self calibration in traditional wireless sensor networks can be found in [41], just varying the sensors radii for better coverage and energy saving.

Hörster *et al.* [42] presents a linear programming algorithm to automatically calibrate the positions and poses of the cameras, aiming at the maximization of the coverage with reduced overlapping. The employment of routable and fixed cameras is also discussed in [17]. Ram *et al.* [34] proposed a heterogeneous methodology that assumes changeable cameras’ field of view, since PTZ cameras are expected to be deployed in the monitored area, along with static cameras. Coverage calibration by managing the camera’s zoom is investigated in [43].

Table 1 summarizes most of the presented works for the problem of optimal camera placement, aimed at the maximum coverage with minimum number of deployed cameras. Most of them compute an optimal solution, but they do not scale and do not deal with connectivity and energy issues, being unfeasible for wireless sensor networks comprised of hundreds or thousands of video-based nodes.

The presented works bring contributions in the optimal placement of cameras to cover an area of interest. Some interesting conclusions can be taken when analyzing their algorithms and experiments.

First, as cameras will be deployed (not nodes with processing capabilities), the algorithms processing are expected to take place in a centralized computer. Hence, such algorithms will face problems of scalability when the number of deployed cameras increases. Other interesting conclusion is about the type of deployed cameras, which can be static or allow changes in their orientations. Static cameras do not allow changing in the coverage area after deployment, requiring offline algorithms for optimal placement calculation that has to be computed before de deterministic deployment. When cameras’ orientation can be changed, online algorithms can manage the coverage adjusting the cameras’ poses along the time.

Finally, the presented works bring different models to represent an area of interest. Most of them consider the monitored field as a 2D area, simplifying the experimental evaluation of the proposed solutions. However, such approach puts the simulated environment miles away from the real world. In a different way, some works investigate algorithms for coverage optimization considering a 3D modeling of the monitored area. The coverage in a 3D area is NP-Hard [23,24], requiring some approximations when dealing with coverage. However, they are more suitable for real-world implementations. All the works described in this subsection cannot be directed applied to solve the coverage problem in video-based wireless sensor networks, but influence investigations in VWSNs.

### 4.2. Optimal Sensor Placement

Optimal placement of cameras is a key requirement in many applications, mainly in controlled and energy unrestricted indoor fields, playing an important role in applications as surveillance, object tracking and recognition in previously known regions (airports, buildings, museums, *etc.*).

The works presented in the last subsection have brought many contributions in visual processing, coverage calculation and overlapping estimation, but they expect that the deployed cameras are wired connected and have a continuous and unrestricted energy supply, making the proposed algorithms unfeasible for video-based wireless sensor networks. Additionally, they consider the deployed cameras with restricted or even inexistent processing capabilities.

Real-world video-based sensors will be constrained in energy and processing resources. Moreover, they will communicate using a low-power wireless link, in an ad hoc manner. Such features turn the optimal placement of video-based sensors more challenging when compared with the works discussed in the previous subsection.

In fact, VWSNs will often be randomly deployed, with sensors scattered in the monitored field. However, such sensor networks can also follow a deterministic deployment, keeping energy preservation and connectivity maintenance.

Younis *et al.* [22] brings a survey of many determinist deployment strategies for traditional wireless sensor networks. Even considering nodes with ominidirectional sensing, the discussions taken in [22] can assist in the deterministic deployment of video-based sensors, with some adaptations. An interesting analysis of the deterministic deployment approaches presented in [22] is the classification of the deployment strategies, which can focus the coverage, the data fidelity, the network lifetime, the communication delay or the connectivity among the sensors. The sensor role to the network, considering sensing and communication, are also employed in the analyses of the deployment strategies.

Oasis *et al.* [21], based on many works presented in the previous subsection, propose centralized offline algorithms to calculate the optimal placement of video-based sensors to cover a monitored field, also addressing energy and connectivity issues. The authors argue that determinist deployment is very suitable for many VWSN applications, since random deployment can result in sensor harm (e.g., after an air drop) and/or suboptimal coverage of a monitored field. Hence, a deployment plan could be used by an engineer to precisely deploy video-based sensor and base stations, potentially prolonging the lifetime of the network and reducing the number of necessary nodes to cover an area of interest.

The work presented in [21] employs an ILP algorithm to compute an optimal placement configuration. The overall solution indicates desired sensor parameters, which are sensing range, field of view and orientation, as well as the number and location of base stations. Different number of deployed sensors and base stations are investigated in experiments, aiming at the reduction in the number of sensors required to monitor an area of interest, and consequently the final cost of the VWSN.

Han *at al.* [44] also proposed solutions for optimal coverage of wireless directional sensors, when sensors can be precisely placed in any location within a monitored field. The investigation conducted in [44] also tries to discover an optimal number of sensors to be deployed, but consider the connectivity of the sensors as the main aspect to be preserved and optimized. Different algorithms are proposed and investigated on experiments. Additionally, deployment patterns are proposed, considering the relation of the FoV of deployed sensors and the resulting covered area and coverage density.

The experimental results in [44] show the relation of the sensing radius, the transmission radius and the required number of deployed sensors for optimal coverage. In the experiments, considering all the proposed algorithms, the number of required sensors decreases when the sensing radius or transmission radius increases. The rate of such decreasing defines the performance of each of the proposed algorithms.

Both works investigate the problem of reducing the number of sensors nodes required to cover a monitored field, using deterministic deployment. They consider different approaches and algorithms, but achieve similar results regarding the number of required sensors and the number of placement sites. Sections 5 and 6 survey additional works aimed at coverage optimization when sensors are randomly deployed.

An interesting and little explored example of deterministic deployment in VWSNs is the use of video-based sensor placed on trees in a forest to monitor and track wild animals. An optimal placement can be calculated, but the lack of infrastructure imposes energy constraints and wireless *ad-hoc* communication requirements. It is an interesting and very recent area of investigation, since most of works in literature are focused in randomly deployed video-based wireless sensor networks or optimal placement of resource-unconstrained cameras.

## 5. Coverage in Random Deployment

Wireless sensor networks were initially envisaged as a technology that would enable sensing applications in regions with limited or absent infrastructure, as well as in wide, hostile or even hard-access areas, employing low-cost sensors. For most applications, sensors are expected to be randomly deployed, since it is easier and less expensive for large wireless sensor networks, and may be the only feasible option [45]. The same is true for video-based wireless sensor networks, but the directional sensing model imposes additional challenges that demand new researches in coverage maintenance and energy preservation.

In order to compensate the lack of exact positioning in random deployment and improve the fault tolerance, nodes are typically deployed in excess, with more sensors deployed than required when compared with the optimal placement [9]. The resulting network will be composed by many redundant nodes, which can be used to save energy as long as maintain coverage and connectivity. The high deployment density can also allow the reduction of the communication range, resulting in energy saving [46].

Rahimi *et al.* [47] argue that a large number of low-resolution battery-operated video-based sensors are a better solution for occluded environments than few high-resolution sensors. Indeed, occlusion is a frequent question in real-world environments. The discussion taken in [47] enforces the using of a large number of randomly low-cost video-based sensors, potentially prolonging the lifetime and coverage of the deployed network. In fact, in typical wireless sensor networks, the number of scattered sensors will be large, with high deployment density [45,48].

Deployment of wireless sensor networks is a crucial issue that impacts network coverage and connectivity. Considering that the monitored field can be wide, hostile or even hard to human access, nodes could be massively distributed from airplanes, rockets or missiles [14], resulting in a large number of unsupervised sensors scattered along the target area. An interesting approach is to improve the initial configuration of the deployed nodes, using strategies as redeployment and mobile nodes [49,50]. Additional complexity of moving nodes and difficulties in access the monitored field has to be properly considered when planning the deployment and post-deployment strategies [21].

The purpose of a VWSN is to monitor a scene or targets, which can be statically positioned or moving across the monitored field. How such monitoring will be performed can guide the deployment of the sensors. Cardei and Wu [9] define three types of coverage: the area coverage, the point coverage and the barrier coverage. Area coverage is the most usual approach, where an area of interest has to be monitored. If the objective is to cover a set of points, sensors could be deployed near of the targets. At last, the barrier coverage aims to avoid undetected penetration through the conceptual barrier formed by the sensors. Additionally, Chow *et al.* [51] investigated the problem of angle coverage, aiming at the reduction of energy consumption by preventing transmission of redundant images, while preserving the network coverage.

Random deployment will be usually performed with no previous knowledge of the monitored field, although the previous knowledge of the targets to be monitored is a conceivable deployment parameter. Nevertheless, some approaches consider the previous knowledge of the region where sensors will be randomly deployed. In [52], the proposed deployment algorithm uses a configuration file containing information about the number of deployable nodes, the length and width of the deployed area, the location of the sink, the obstacles, among others.

### 5.1. Node Localization after Deployment

After random deployment, sensors can be placed anywhere in the monitored field, and their location and deployment density cannot be previously known. Hence, it is expected that sensors discover their current location, since it is required for many applications as pattern tracking and surveillance, as well as for many algorithms in coverage optimization, connectivity maintenance and energy preservation. A localization solution has to be applied since the connectivity of the nodes (what nodes are accessible by what nodes) and their coverage (what regions are covered by each sensor node) have to be properly discovered, along with the spatial coordinates of the nodes. Also, most applications in video-based wireless sensor networks rely on the knowledge of sensor positions and the current poses of the cameras. The localization of randomly deployed nodes is also defined as a problem of topology extraction after deployment.

Some localization algorithms for traditional wireless sensor networks can be found in [53,54]. As video-based sensors work following a directional sensing model, the current direction of each sensor is also unknown, requiring specific algorithms for node localization.

Pescaru *et al.* [50] classify the localization solutions into fine-grained and coarse-grained. The first group is based on direct parameters such as time and signal strength, while the last uses proximity to a reference point, indirectly calculating the localization of the nodes. As already expected, connectivity relationships in video-based wireless sensor networks can be discovered using traditional algorithms from WSN, but the coverage of the network has to use algorithms adapted to the directional sensing model. For example, GPS (Global Positioning System) cannot be employed for coverage discovery in VWSN due to the lack of information about the orientation of the cameras, besides the cost and energy waste [55].

Typically, the localization algorithms can be processed in a centralized or distributed fashion. Centralized algorithms are processed in the sink or in a central server, saving energy by avoiding additional processing in the nodes. Also, as the sink is expected as a resource unconstrained device, complex algorithms can be easily executed. The drawback is the low scalability of the centralized algorithms. For distributed algorithms, the localization discovery is processed by each node, in an independent way, using neighborhood information. Distributed algorithms scale better, but demand processing in the wireless sensor nodes.

For node localization in video-based WSNs, most solutions regard the overlapping area of the sensors’ FoV, employing algorithms from the computer vision area to estimate the position of the nodes. In [56], images from different nodes are processed in a central server, which computes field of view superposition. The proposed centralized algorithm calculates parameters like coordinates translation, rotation angle and scaling factor. These parameters are then diffused throughout the entire network, using a protocol specified in that work. The final node localization is based on estimation of these parameters between each pair of neighbor nodes. The work in [57] is an extension of [56], using a mean shift based solution and image registration to improve the calculation of the previously presented parameters.

Lee and Abhajan [58] presented four distributed localization methods for visual sensor networks. In the first method, the observations of neighboring nodes are utilized for node localization, identifying the overlapping among the retrieved images. The other three methods are based on simultaneous observation of a moving target, which can have an arbitrary motion, a constant velocity or know their own coordinates. Experimental results are useful for comparison of these approaches.

In [59], a moving target is also used to discover the position of the cameras. That work defines the Simultaneous Localization and Tracking (SLAT) problem, where the poses of the cameras are estimated along with the trajectory of the moving target, employing a distributed online algorithm.

Localization of directional sensors is also investigated in [60]. The proposed method automatically identifies the overlapping areas of cameras’ field of view to estimate the nodes locations and directions, and allows online update of the information about the network topology, if some change occurs. It can also handle heterogeneous video-based sensor types. The redundancy of the cameras’ views is exploited to achieve better performance than similar algorithms.

Devarajan and Radke [61] presented a distributed algorithm for localization and self-calibration of randomly deployed video-based wireless sensor networks. The sensor network is modeled using two undirected graphs. The first is the communication graph, representing the ad-hoc wireless communication between the nodes. The second graph represents the vision relationship between the cameras, where two nodes are associated if and only if they view the same scene or object (even under different perspectives). The neighborhood in the vision graph is used for calibration. The work presented in [62] continues this investigation, bringing more details and experimental results.

A distributed solution for node localization considering ominidirectional viewing of video-based sensors is presented in [63]. This work specifies a linear iterative algorithm to estimate camera’s position and orientation, using overlapping areas. In [64], information about camera’s field of view is used for 3D node localization, dealing with different methods to obtain the location of the nodes. It also briefly discusses a solution for distributed localization of a large number of video-based sensors, since the experiments for node localization in most works only regard few deployed sensors.

### 5.2. Coverage Algorithms

Many works have investigated the optimization of positioning of cameras and (more recently) sensors considering deterministic deployment, as presented in the last subsection. When sensors are randomly deployed, the coverage can be also optimized, based on the positions, orientations and numbers of the sensors. The algorithms for optimal camera/sensor placement following deterministic deployment are a reasonable basis for coverage optimization in randomly deployed video-based wireless sensor networks, with some adaptations.

When video-based sensors that cannot change their current orientations are randomly deployed in a monitored area, the covered area will be defined by the current positions and orientations of the sensors just after the deployment. In this case, an algorithm can only compute redundant nodes in order to try to prolong the network lifetime, since the covered area cannot be changed (but can become depleted over time).

If the current orientations of the sensors are changeable, algorithms can compute optimized orientations for a maximized covered area with a minimum number of active nodes. Figures 2(b,c) show a graphical representation of how changing cameras’ orientation can improve coverage and produce redundant nodes.

Ai and Abouzeid [45] present two centralized and one distributed algorithm to calculate the initial orientations of directional sensors in order to cover as many targets as possible, activating the minimum number of sensors. The calculated orientations are used to change the directions of active video-based sensors, letting inactivated sensors to replace failure or energy-depletion, potentially prolonging the network lifetime. This is defined by the authors as the Maximum Coverage with Minimum Sensors (MCMS) problem, which can be solved by two centralized (ILP and greedy approaches) and one distributed greedy algorithm.

The proposed algorithms are verified by experiments. As expected, the ILP centralized approach performed better (larger coverage with less active sensors) than the centralized and distributed greedy algorithms. However, ILP algorithms demand more energy and processing resources than greedy algorithms, and centralized solutions do not scale, putting the proposed distributed greedy algorithm as a better solution for typical VWSN.

The authors also noticed that increasing the number of deployed sensors linearly increase the coverage ratio and the number of active nodes, until the deployed sensors reach a threshold. Upon this value, the number of activated nodes increases slowly or even decrease, whereas the coverage ratios continuously increase. This threshold is a function of the number of targets in the modeled experimental environment, but attests that the deployment of many nodes can potentially produce redundant nodes.

Cai *et al.* [65] also considered the random deployment of sensors with changeable orientations, but focus on the activation of nodes for target monitoring. The proposed solution intends to cover all the targets defining the orientations each sensor has to assume, deactivating redundant nodes. This is defined as the Multiple Directional Cover Sets (MDCS) problem. The directions of the sensors are organized into non-disjoint subsets (cover set), allowing sensor to participate in multiple sets. For example, one cover set can be composed by three sensors, each with a particular FoV orientation. The same three sensors can create a different cover set, just changing their orientations. At each time, one cover set (comprising one or more sensors with their FoV orientations) is activated. If a sensor is not in the currently activated cover set, it goes to sleep state.

To calculate the cover sets, three centralized algorithms are proposed, based on linear programming and heuristics. One of them, the “feedback algorithm”, aims to reduce the number of cover sets, also reducing the time spent in transition between different cover sets, potentially reducing the time interval that the targets are not covered. The experimental results compare the performance of each of the proposed algorithms considering different number of sensors and targets. Since each cover sets must cover all the targets, the experiments are mainly concerned with the prolonging of the network lifetime by exploiting the existence of redundant cover sets.

Both the previous works optimize coverage and produce redundant nodes that can be used to prolong network lifetime while preserving coverage. Section 6 explores the relation among coverage, connectivity and energy preservation, revisiting the references [45] and [65].

In randomly deployed wireless sensor networks, some regions in the monitored field can be sparsely covered or suffer with high occlusion. For example, a camera’s FoV can become useless if its current orientation results in the coverage of a wall. However, if the orientation of that camera can be changed, the camera may view a useful area of the monitored field. The work in [66] proposes a distributed method to change the orientation of wireless nodes to minimize the effects of occlusion. Each node independently discovers its neighbors and analyzes obstacles and overlapping regions. According to discovered values from the neighborhood, the nodes can automatically adjust the orientation of the cameras, changing their field of view.

An interesting result presented in [66] is that for highly-occluded fields, many low-resolution cameras are a much better solution than few high-resolution cameras, as also attested in [47]. Moreover, it is also posed that the increasing in the deployment density does not expand coverage in the same proportion, resulting in increased overlapping. A similar conclusion is obtained in [67].

Cameras with angular mobility are also investigated in [68]. Visual sensor networks with sensor nodes equipped with cameras having angular mobility can dynamically manage a covered area, avoiding blanket spaces and undesired overlapping. The proposed distributed algorithm aims to find the direction of least density of neighbors.

### 5.3. Coverage Metrics

After random deployment, video-based sensors will be scattered along the monitored field, with unpredicted positions and orientations. Some algorithms can be employed to improve the coverage of the deployed network, but the final outcome depends on the sensors configuration after deployment and the “performance” of such algorithm.

Frequently, it will be desired to know the quality of the calculated coverage. Many works have been concerned with coverage metrics, for both traditional WSNs and VWSNs.

In [69] the level of coverage and connectivity of a deployed traditional WSN is measured, being classified in three different groups: full coverage with connectivity, partial coverage with connectivity and coverage with constrained connectivity. The author argues that coverage without connectivity is meaningless in wireless sensor networks, since the collected data cannot be retrieved from an offline node. The same idea can be true for video-based wireless sensor networks.

Pescaru *et al.* [50] defined a reasonable metric to measure the deployment quality in terms of coverage, considering video-based sensors. In that work, the Node Area (NA) is defined as a circle centered in the node, and the Network Coverage Area (NCA) as the union of all NA. The relevant sensing area of each node is only a sector of this circle, representing an intersection of the field of view and the node area. The resulting Deployment Coverage Quality (DCQ) is the ratio between the sum of all relevant sensing areas (NRSA) and the NCA. Experimental results presented in [50] showed that the DCQ increases when the number of deployed nodes also increases. A similar metric is also described in [70]:

$$\mathrm{DCQ}=\frac{\mathrm{NRS}A}{\mathrm{NCA}}$$

Another metric that can be used to measure coverage after deployment is the K-coverage, which says that every point in the deployed region is within the coverage ranges of at least K sensors [7]. For example, if a deployed region is 3-coverage, every point is covered by at least three sensors, and the failure of one or two nodes sensing the same region still maintain that particular region covered (but not necessarily connected). Wan and Yi [71] investigated the probability of a region to be K-covered by changing the sensing range of isotropic sensors. The work presented in [72] argues that when the communication range is at least twice the sensing range, a K-covered network result in a K-connected network. All these works bring contributions that can be applied to video-based wireless sensors networks, with some restrictions.

Liu *et al.* [73] proposed the directional K-coverage (DKC) to measure the coverage quality in VWSN. The authors verify that, for randomly deployed video-based sensors with a uniform density, it is very difficult (if not possible) to guarantee 100% coverage. As a result, DKC is defined as a probability guarantee. The DKC is a function of the sum of the probabilities of the video-based sensors’ views of points of the monitored field, considering the number of deployed nodes (N) and the K factor (number of sensors covering the same region/target). The p_C_ is the probability of coverage of a target by the camera C, considering the field of view of the camera, the angle between the target and the camera direction, as well as the sensing radius. The final probability of coverage is given by:

$$P=1-\sum_{m=0}^{k-1}C_{N}^{m}p_{C}^{m}{\left(1-p_{C}\right)}^{N-m}$$

This metric can be useful to validate a deployed VWSN regarding applications that require more than one camera viewing the same target, as face orientation detection [74].

Other metrics can also measure the coverage in video-based wireless sensor networks. In [36] is presented the *FoV maximal breach path*, a centralized-computed metric based on the distance from any sensor to the closest observed point. For tracking application, the monitored targets will pass by this observed point, making this metric suitable for such applications. As an alternative, Pescaru *et al*. [20] defined a distributed light algorithm to compute the resulting coverage in densely deployed video-based wireless sensor networks, which is an approximation of the *FoV maximal breach path*. The proposed algorithm is defined as *FoV closest path*. The experimental results presented in [20] show that the proposed metric performs better when execution time and resources are constrained.

The metric presented in [50] is easy to compute and can be used as a fundamental metric for coverage in VWSN. When it is desired a more precise metric that considers every target covered by a defined number of cameras, the metric presented in [73] should be used. Applications as visual surveillance will benefit from the metric described in [20]; the proposed distributed algorithm can measure the coverage quality by indicating regions with low or inexistent coverage.

## 6. Connected Coverage and Energy Preservation

For video-based wireless sensor networks, coverage, connectivity and energy consumption are interrelated issues that have to be properly handled. The coverage is related to the sensing of a monitored field and the required covered areas depend on the application requirements. The coverage of a single sensor is limited, so wireless collaboration with other sensors is expected to allow coverage over large areas. So, the connectivity becomes a key aspect since offline nodes are useless. In short, it is desired a connected coverage of a scene or target. At last, sensors in VWSNs are expected to be energy constrained [14,15]. As the energy resources is directly related with the network lifetime, energy consumption in processing and communication has to be reduced in each node. A very used approach for energy preservation is to minimize the number of active nodes, employing redundant nodes to replace sensors with depleted energy. This approach put together coverage, connectivity and energy, since turning off nodes usually also deactivate their sensing and communication functions.

**Figure 4.**
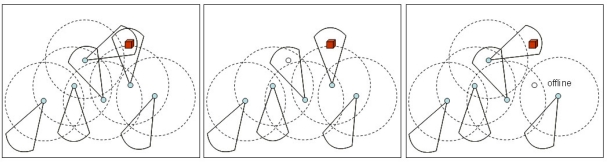
Coverage, connectivity and redundant nodes. **(a)** Network configuration after deployment; **(b)** A redundant node is sent into sleep mode; **(c)** bad selection of the redundant node to enter the sleep mode.

Figure 4 presents a very simple example of a video-based WSN in three different configurations. The dashed circle around the sensors represents the ominidirectional communication range, and the directional sensing capability is defined as a sector of a circle. In Figure 4(a), all sensors are active, resulting in redundant viewing of a target. Energy can be saved turning off redundant nodes, following, for example, the configuration presented in Figure 4(b). Note that redundancy depends on the nature of the application, since each video-based sensor has probably a unique view of the monitored field. For a surveillance application, for example, distinct viewing could bring the same information for the application processing, resulting in redundancy. The deactivation of redundant nodes can produce offline nodes or subnetworks, as happens in Figure 4(c). Such configuration should be avoided if an option that preserves connectivity is available.

As attested before, video-based sensors will be typically deployed in excess, in a random fashion [45,50]. The resulting network will be probably composed by many redundant nodes, which can be used to prolong the network lifetime. Additionally, redundancy can be used to compensate node failure and preserve coverage of the monitored field. For wireless sensor networks, failure of nodes due to lack of power resources or even physical damage (in harsh environments) can be a constant.

Accessing a sensor node for replacement or battery recharge may be impossible due to the nature of the target area. In fact, many works propose algorithms and protocols for energy saving based on redundant nodes turn off because it is expected, for a typical video-based wireless sensor network, that the area of interest will be covered by many nodes. In fact, the sensor network should last for much longer than the lifetime of individual nodes [75].

Energy consumption happens at least in hardware functioning, local processing, sensing functions and communication. Although almost never considered by most of the works, even simple operations as node turning on and off can consume energy [76]. In fact, activation and deactivation of hardware components, as well as the transition between idle and sleep mode, may require considerable energy and may take substantial time [77]. Such considerations sometimes make the experimental environment a weak approximation of real-world implementations.

### 6.1. Saving Energy by Redundant Nodes

In a wireless sensor network, a node can be sensing, relaying messages or be in an idle state. Since even an idle communication-receiving circuit can consume almost as much energy as an active transmitter, idle nodes should be sleeping [78]. However, a certain amount of active nodes should exist to ensure a desired level of coverage at all times.

The coverage of a sensor network depends on the number and the arrangement of sensors. Additionally, for video-based wireless sensor networks, the directions of the sensing unit (cameras) also play an important role. Nevertheless, while moderate loss in coverage can be tolerated by some applications, loss in connectivity can be fatal. The adopted solution has to balance these two issues, considering node deployment and redundancy, application requirements and current energy resources of the nodes.

A survey on the routing techniques and protocols for wireless sensor networks, including energy aware routing, can be found in [79]. Halawani and Kahn [80] also presented a survey on techniques, algorithms and protocols for network lifetime enhancement.

When designing a mechanism to prolong network lifetime by using node redundancy, as far as preserving coverage and connectivity are concerned, three fundamental questions must be answered [9]. The first question is to decide which rule each node should follow to determine whether to enter sleep mode. Possible answers are remaining energy resources, energy already spent, the role in the sensing functions (e.g., a privileged position or a sensor in a sparsely covered area) and the nature of the sensor. The second question is when nodes should make such a decision. Nodes can employ a random counter, they can follow a predefined schedule or even monitor their current energy resources and targets covering. At last, the third question is for how long a sensor should remain in the sleep mode. The designed solution can put nodes to sleep for a fixed or randomized time, as well as it can expect that nodes probe the neighborhood looking for failed or energy depleted nodes, or even wait external events. All these questions must be answered when designing algorithms for coverage optimization in video-based wireless sensor networks.

A fourth question could be stated considering the nature of the sleeping mode. In fact, a sleeping sensor could be in a very low-energy operation, without sensing functioning but still allowing communication. Other approach is to turn off the sensing and communication units, executing locally only a small algorithm to count the time until the node reactivation. Other option is to allow periodical sensing, but only under a very small frequency.

Connectivity preservation, coverage maximization and energy saving can be performed in different ways. When dealing with real-world environments, however, some of these aspects can be prioritized over the others. It impacts the election of nodes to routing or sensing functions. For example, if a region of the monitored field can only be viewed by a unique sensor node, for example, it should not be elected for routing, leaving it for sensing function. Perillo and Heinzelman [12] proposed a protocol that preserves nodes with higher importance for the sensing application, electing them for sensing instead of routing. It is particularly relevant for sparsely covered areas, which can be created after deployment or after many nodes deaths or failures. On the other hand, some approaches can prejudice the coverage on behalf of the prolonging of the network lifetime. It could be performed by reducing the number of active sensors even if uncovered areas appears, increasing the number of redundant nodes. Such approach, of course, should comply with the application requirements.

A trivial but very important aspect of using redundancy to save energy is to how to find redundant nodes. In traditional wireless sensor networks, the ominidirectional sensing nature indicates that neighboring nodes are likely to collect similar raw data. When considering directional sensors, this is not always true, as close video-based sensors may not retrieve the same visual data due to theirs orientations or occlusion. Two nodes are neighbors if they have overlapping field of view, and this can be checked using many algorithms from computer vision area. For example, Bai and Qi [81] define a semantic neighbor selection of directional sensors using comparisons of the retrieved images. The proposed algorithms allow the identification of neighboring directional sensors, which is necessary for energy preservation algorithms considering redundancy.

### 6.2. Algorithms for Coverage, Connectivity and Energy Preservation

In the last decade, many works have addressed coverage, connectivity and energy preservation in traditional sensors networks, exploring redundant nodes. In [78], each node in a WSN running the proposed SPAN algorithm has to periodically decide if it sleeps or stays awake as a node coordinator, participating in the forwarding backbone topology. Cerpa and Estrin [82] proposed the ASCENT protocol to also exploit the redundancy among sensors nodes. Each node self-adapts its participation in the ad-hoc network based on the measured operating region. Ye *et al.* [75] proposes the PEAS protocol. This lightweight protocol keeps only a necessary set of sensors working, while putting the rest in a sleeping mode. Along the time, sleeping nodes wake up and probe the local environment to replace failed or energy depleted nodes. For PEAS, the sleeping time is dynamically and randomly adjusted by the nodes, better adapting for high density of deployed nodes.

Due to inherent particularities of visual wireless sensor networks, especially their directional sensing model, algorithms and computational solutions for traditional WSNs may not be feasible for VWSNs. The following works investigate and propose solutions for coverage, connectivity and energy preservation for visual wireless sensor networks, exploring redundancy among sensor nodes.

Reference [45] presented centralized and distributed algorithms to turn off certain redundant nodes for energy saving in VWSNs, maximizing the number of targets to be covered while minimizing the number of active sensors in a instant of time. After a random deployment, not all targets are covered by the deployed sensors. The proposed algorithms calculate the initial orientations of the directional sensors in order to cover as many targets as possible, activating the minimum number of sensors.

In order to achieve scalability, the distributed solution proposed in [45] allows each node to make its own (activation and orientation) decisions based on local information gathered from neighboring nodes. In order to guarantee a trade-off between coverage and network lifetime, the Sensing Neighborhood Cooperative Sleeping (SNCS) protocol is proposed. Employing SNCS, each node continuously alternates between two distinct phases: scheduling and sleeping. In scheduling phase, each node decides independently if it stays active or inactive, taking in account the possible covered targets and remaining energy. The proposed distributed greedy algorithm considers the residual energy of each node, with nodes with more residual energy having a greater priority to become active. The SNCS protocol is employed by nodes to discover the residual energy of other nodes in their communication range vicinity. Such behavior result in a balance in nodes activations, potentially prolonging the network lifetime while preserving coverage.

The SNCS protocol performance and robustness are evaluated by experiments, considering many parameters as packet losses and localization errors. The experimental evaluation is useful in planning the monitoring of a target area, since the number of targets and sensors, as long as the sensing range, impact the final performance of SNCS and proposed algorithms.

The activation and deactivation of nodes can also consider the importance of the sensors for the coverage duty. Pescaru *et al.* [4] proposed an algorithm that turns off the less significant nodes that are redundant, possibly reactivating them if necessary. This algorithm has two phases. Initially, after network deployment, the areas covered by more than one sensor node are identified. The significance of the nodes is evaluated by calculating the percentage of overlapped areas, with the less significant being turned off. In the second phase, the low-energy or malfunctioning nodes are detected. To keep coverage and prolong network lifetime, an active node with energy resource below a threshold try to wake up a neighboring sensor, following a local calculated table containing the nodes and their correspondent significance for the coverage: The most significant sleeping node for that monitored area is awaken. For the proposed algorithm, a node is redundant if the area covered by that node is also covered by at least one more node by a percentage not less than 70%.

The experimental verifications conducted in [4] also investigate the efficiency of the proposed algorithm in a monitored area with a variable number of deployed sensors. It is verified that the coverage area and the number of redundant nodes increase when the number of deployed nodes also increases, as also attested in [45]. The efficiency is measured considering the using of the proposed algorithm with variable parameters and when redundancy is not exploited, in a different way of [45], which focuses on comparisons among the proposed algorithms. The authors in [4] verified, after the experiments, that use of the proposed algorithm increases the coverage when nodes death occurs, exploring redundant nodes.

Both works presented in [45] and [4] benefit from the fact that, typically, the number of deployed nodes are huge, and it is likely that some areas are covered by more than one sensor’s FoV, turning redundant nodes a reality. The efficiency of the strategies strongly depends on the node redundancy. However, such redundancy is exploited in different way by these works. While [45] balance the energy depletion by activating and deactivating sensors along the time, the algorithm in [4] only wakes up nodes when the energy resource of an active node is below a threshold.

For the two works presented before in this subsection, redundant nodes are employed to compensate for energy depletion, though following different approaches. However, the activation of redundant sleeping nodes can happen due to an external event. Istin *et al.* [83] proposed an algorithm to maintain image acquisition even in the presence of dynamic disturbance (as moving obstacles), when there are many deployed sensors. Initially, redundant sensors are turned off, letting only one sensor viewing the monitored field. When the FoV of an active node is obstructed (FoV loss is greater than 70%), the node sensing function is turned off (for resource preservation) and the proposed algorithm identifies an optimized set of redundant nodes that must be turned on to cover the FoV loss. Nodes with sensing functions turned off keep theirs communication functions on, in order to maintain the nodes connected.

The algorithm proposed in [83] is distributed and online, with a direct (but not limited) application in traffic monitoring. When a node detect that its FoV is lost due to a moving obstacle (as a car or truck) entrance in the camera viewing, it indicate such event to its neighboring nodes. To identify the optimal subset of sensors that should be turned on, a cost function was created. This cost function regards the covering of the current FoV loss, the resources available and the potential of covering the FoV losses that neighboring nodes might experience in the near future.

Experiments in [83] verified the loss recovering in the presence of obstacles, studying simulated environments with variable number of cars, speeds and FoV redundancy. In most experiments, the FoV recovery was higher than 40% with the number of cameras ranging from three to five, and higher than 85%, considering the number of cameras from three to 15. It was verified that the coverage loss increases significantly when the number of cars also increases.

The work presented in [83] considers nodes with rechargeable batteries, but expect that energy consumption is kept in minimal levels. A similar research is taken in [84], considering coverage preservation and network lifetime prolonging in an environment with moving obstacles.

Cai *et al.* [65] consider an energy saving strategy based on the monitoring of targets by randomly deployed video-based sensors, with the directions of the sensors organized into non-disjoint subsets (cover set). At each time, only one cover set is activated, letting the remaining in sleep mode. A sleeping cover set is selected to replace the previously activated set, usually due to energy depletion.

Experimental results from [65] compared the three proposed algorithms in function of the network lifetime. In all experiments, the network lifetime increases with the increasing of the number of deployed nodes and the sensing range. However, the network lifetime decreases when the number of targets to be monitored increases. Other interesting result is that the increasing in the number of directions a sensor can assume directly impact in the network lifetime. In short, more directions means increased network lifetime.

A scalable solution to reduce energy consumption in VWSNs is also proposed in [85]. Power management policies are defined, each one defining rules to send nodes to sleep state, considering the importance of the node for the monitoring and/or probable redundancy. Such importance is measured considering the monitoring of a moving target; if the sensor cannot view the target for a period of time previously defined, it goes to sleep mode. Nodes return to active mode after a sleep time has elapsed. A proposed alternative is to regard redundancy by the transmission of messages that indicate the views of neighboring nodes. Nodes use this information to decide if they should turn themselves off.

Table 2 summarizes the presented algorithms and protocols to prolong the network lifetime by saving energy in video-based wireless sensor networks, while preserving coverage and connectivity when there are redundant nodes.

The success of the adopted solution also depends on video-based sensors hardware. For example, Rahimi *et al.* [86] employs hardware that can be put in standby mode. In this mode, sensing functions are deactivated, but communication is still possible. These behaviors create a separate treatment of the network aspects and the image sensing. The energy consumption when hardware functions are active and the energy preservation in sleep mode have to be also properly considered [80].

Using redundancy to prolong network lifetime should not prejudice the coverage requirements of the applications. In the previous section, some metrics to measure the coverage were presented. Those metrics could be applied to dynamically verify the coverage when the network configuration is changed due to deactivation of sensor nodes.

## 7. Other Relevant Issues

Previous sections have surveyed many works comprising different issues of the coverage problem in video-based wireless sensor networks. In this section, other relevant investigations will be surveyed, considering aspects as camera selection, mobile sensors, architectures for video-based sensors and directional communication.

As each video-based sensor has a direction and different field of views can overlap, images from distinct viewpoints can be obtained from the network by combining the retrieved visual data from the monitored field, just selecting the appropriated cameras. Soro *et al.* [87] presents a method to reduce energy consumption and data redundancy by selecting which parts of retrieved images from each camera should be sent to the sink in order to reconstruct a desired viewpoint, considering 3D field of view. For the proposed solution, two distinct methods are evaluated. The first one is based on the minimum angle between the direction of the desired view and the direction of the camera. In the second approach, cost metrics are applied to measure the camera’s importance in the monitored area. This approach also considers the remaining energy of the sensor nodes. The proposed solution is an extension of the work presented in [88], which regards 2D viewing and uses no metric to evaluate the camera importance for scene viewing.

The authors in [89] employed a look-up table with information about cameras’ field of view. This table is then used to select the cameras that are more suitable to obtain the desired image from a specific location, using the same angle calculation utilized in [38].

The camera selection problem for target localization is also investigated in [90,91], using a 2D model with cameras placed horizontally around a room. The proposed algorithms for camera placement and selection use a linear estimation and the concept of mean squared error.

The sensor cameras can be moving or have movement capability, give rise to new challenges for the VWSN coverage. McCurdy, *et al.* [92] proposes a system for continuous and probably wide coverage by employing head-mounted cameras in personnel who (typically) moves across an area of interest. An algorithm for switching viewed images in a soft way is also presented. The drawback of this solution is that coverage cannot be predicted, and blank areas can be created along the time. Lee *et al.* [93] designed a VWSN with a mobile sink. Authors argue that such configuration can improve coverage, with reduced energy consumption. The work presented in [93] also expects the using of solar rechargeable energy batteries, as an option to prolong the network lifetime.

A modeling environment for virtual simulation of camera placement is presented in [94]. Real-world coverage constraints, as obstacles and camera viewing (field of view, focus, resolution, *etc*.) are modeled in the virtual world. Authors argue that changing in camera positioning is easier in the developed virtual environment than in real-world, improving the analysis of the coverage of a set of cameras: as the virtual environment tool calculates the mutual viewable volume for the cameras, users can see if a particular object is in the coverage area of the deployed cameras. In [72], users can “virtually” navigate through a region covered by a VWSN, specifying a viewpoint that changes with time. For each viewpoint selected, the appropriate cameras are selected, retrieving the most appropriate image. Simulations are performed regarding different metrics, as viewing angle and coverage, for methods with different energy saving concerns. The algorithms and simulations scenarios in this work consider a 2D modeled environment.

Feng *et al.* [95] presented architecture for low-power video-based sensors, considering issues as coverage, energy consumption and communication. Reference [96] employs heterogeneous sensors to achieve the application requirements. Due to the variety of sensors available, including sensors with video capabilities, the proposed architecture allows different levels of quality and costs. The proposed multi-tier architecture is argued to be a better solution than networks composed of only one sensor type. A similar investigation is taken in [97].

Video-based sensor networks employ a directional sensing model to retrieve visual data from the environment, but the wireless nodes typically communicate following an ominidirectional model, similar to the communications in traditional WSNs. The work presented in [98] investigates directional communication, bringing sensing and connectivity to the same conceptual scope. In that work, while wireless nodes receive data in an ominidirectional way, data sending occurs only in the node’s field of view direction. In the proposed model, two nodes can directly communicate only if one is in the directional communication area of the order node. Strategies for communication checking and repairing are also discussed. According to the authors, this particular type of communication imposes new challenges for the prolonging of network lifetime and connectivity maintenance.

## 8. Open Research Areas

Many issues are related to the coverage problem in traditional wireless sensor networks. When video-based sensors are employed, new challenging requirements have to be properly considered, in order to obtain a reasonable performance with minimal cost.

The coverage in networks of video-based ad hoc sensors comprises a lot of aspects that have been addressed in several recent works. However, many issues are still uninvestigated, signalizing promising research areas. Based on the surveyed works presented in the previous sections, some open research areas can be envisaged.

Most of the works considering video-based wireless sensor networks make unrealistic assumptions, as linear cameras’ field of view, homogenous illumination and planar deployment regions. For example, the works presented in [45,65] assume that directional sensors are homogeneous, with the same ominidirectional communication range. Additionally, many works also model the monitored field in only two dimensions. Such simplifications are relevant when measuring the performance of algorithms and protocols, but they can result in an unreal analysis of the proposed solutions. Future works should address coverage in more realistic environments. For example, a still very little explored research area considers illumination variation in the monitored field, since retrieved images and videos could be affected by low or high light intensity. A promising approach could use the current sun position to activate or deactivate sensors, benefiting from light source direction. Other interesting investigation is to regard sensors equipped with a flash-type camera. The camera’s flash could be used to compensate the lack of illumination of some regions of the monitored field or even be used to allow video monitoring during the night using conventional cameras. In other type of investigation, the relief of the monitored field could be considered when dealing with the coverage of the deployed networks. Sensors in a higher floor could view different targets than other sensors, and it could be an additional parameter for algorithms that use redundancy to save energy.

The random deployment of wireless sensor can be performed in different ways. For a wide and hard-access monitored field, airdrop seems to be the most feasible option. But when video-based sensors are dropped, we should be concerned with the orientation of the embedded cameras. For example, sensors dropped from an airplane should not land having it camera directed to the ground or to the sky (unless required by the application), since it could become useless for sensing functions. Additionally, future research should also investigate how video-based sensors can better survive an airdrop. Considering that cameras’ lens are typically fragile, some mechanism as a parachute should be employed, avoiding harming when sensors reach the ground. However, depending on the weather condition, wind can blow sensors away from the monitored area. All these issues have to be properly investigated.

Most experiments related to the coverage problem only contemplate the deployment of dozens of sensors. However, real-world implementation can require hundreds or even thousands of sensors. Such demand should be properly investigated, since the performance of many proposed algorithms could be affected by a large number of deployed sensors.

Other interesting research area employs audio and video sensors to retrieve multimedia data from the monitored field. Few works investigate heterogeneous multimedia sensors, and many details related to audio and video coverage are still open. The directional coverage could also be extended to incorporate other types of sensors, as infrared.

Mobile nodes can become a common approach to monitor moving targets or even to “walk” in devastated regions. Sensors could be deployed in mobile robots or rescue dogs, resulting in a frequently changeable coverage of the video-based sensor network. Directional coverage in such environments can become very challenging, demanding more complex and robust solutions to deal with mobile video-based sensors. Along with mobile nodes, future works could better investigate nodes statically positioned following a deterministic deployment.

Ma *et al.* [98] investigates a directional model for sensing and communication. This approach is still few explored, and additional works could further discuss the benefit and drawbacks of directional communication and coverage. Other open research areas may emerge with future investigations of the coverage problem in video-based wireless sensor networks, demanding additional efforts from the research community.

## 9. Conclusions

In short, the coverage problem is concerned about how well an area of interest is covered by wireless sensors, and, for densely deployed areas, how the lifetime of the network can be prolonged by switching sensors on and off, maximizing the time since the first lack of sensing occurs in any subspace of the monitored field. When sensors are equipped with a camera, the resulting coverage follows a directional sensing model, requiring properly solutions to address the new challenging issues imposed by these video-based sensors networks.

In this paper, many works comprising the coverage problem in VWSNs have been surveyed, considering deterministic and random deployment, coverage metrics and energy efficiency, among other topics indirectly related to the coverage problem. Some promising open research areas have been also presented, indicating a possible direction for future works.

## Figures and Tables

**Table 1.** Algorithms for optimal camera placement.

**Optimal Placement Solution**	**Algorithm Approach**	**Short Description**
Mittal and Davis [31]	Probabilistic visibility analysis	Optimal placement of cameras considering occlusion created by dynamic obstacles.
Erdem and Sclaroff [19]	Binary Optimization	Suitable for planar regions, following task-specific requirements.
Hörster and Lienhart [32]	ILP	The monitored field is modeled as a 2D grid. Optimal placement considers cost restrictions.
Hörster and Lienhart [33]	BIP / Heuristics	Proposes both an exact and an approximated solution for optimal placement.
Ram *et al*. [34]	BIP / Based on performance metrics	Optimal placement of multimedia camera/sensors, considering heterogeneous nodes.
Zhao *et al.* [35]	BIP	The monitored field is modeled in 3D. The authors expect self and mutual occlusion.
Zhao and Cheung [36]	BIP	Grid-based optimal camera placement. Also discusses visual tagging.
Couto *et al.* [38]	IP	Model the art gallery problem using ominidirectional cameras.
Gonzalez-Barbosa *et al.* [40]	ILP	Employs directional and ominidirectional cameras in a hybrid way for coverage optimization.
Hörster and Lienhart [42]	BIP	Automatically calibrate camera directions for coverage maximization with minimum overlapping.

**Table 2.** Algorithms for coverage maintenance and energy saving in VWSNs.

**Algorithm**	**Short Description**
Ai and Abouzeid [45]	The SNCS protocol utilizes the residual energy of each node as a priority for putting nodes into sleep mode. Sleeping nodes can become active when theirs energy resources surpass the residual energy of current active nodes.
Pescaru *et al.* [4]	The proposed algorithms turn off less significant redundant nodes. Each active node evaluates its energy and if it is lower than a threshold, a redundant neighbor node in sleep mode is turned on.
Cai *et al.* [65]	Define subsets of sensors to cover a target, with individual sensors participating in one or more cover sets. Only one subset is activated at any time, saving energy by deactivating the remaining sets.
Istin *et al.* [83]	The nodes that detect FoV loss inform the neighboring nodes. Based on the answer, the node identifies the optimal cameras that should to be turned on. After the obstacles passes by and the original FoV is restored, the original node informs the neighboring cameras that attended the previous request that they should turn themselves off.
Zamora *et al.* [85]	Nodes that do not view the monitored target go to sleep mode, self activating after a fixed sleep time. Nodes can also exchange messages indicating the current and past views of the cameras. Such information is used to send nodes into sleep mode, potentially prolonging network lifetime.
